# Extramedullary Hematopoiesis in Uterine Leiomyoma Associated with Numerous Intravascular Thrombi

**DOI:** 10.1155/2014/957395

**Published:** 2014-03-06

**Authors:** Xiaoyan Cui, Deniz Peker, Heather O. Greer, Michael G. Conner, Lea Novak

**Affiliations:** ^1^Department of Pathology, University of Alabama at Birmingham, P210 West Pavilion, 619 19th Street South, Birmingham, AL 35233-7331, USA; ^2^Department of Obstetrics and Gynecology, University of Alabama at Birmingham, Birmingham, AL 35233, USA

## Abstract

We report a case of extramedullary hematopoiesis (EMH) in uterine leiomyoma and associated numerous intravascular thrombi. A 29-year-old nulliparous female presented with heavy vaginal bleeding and a hematocrit of 22%. No bone marrow biopsy has been performed. She had a history of uterine leiomyomata and menorrhagia for a year. A transvaginal ultrasound confirmed the presence of a uterine leiomyoma. The patient was treated conservatively with oral contraceptive pills due to desire for fertility. However, she continued to have heavy vaginal bleeding and developed bilateral upper extremity deep vein thrombosis and multiple superficial vein thromboses after two months. An exploratory laparotomy with uterine myomectomy was performed. Gross examination of the specimen revealed a single nodular mass measuring 10.0 × 9.5 × 7.5 cm with a white-tan swirling cut surface. Microscopic examination revealed benign smooth muscle consistent with leiomyoma and numerous intravascular thrombi both with areas of EMH. Immunohistochemical stains confirmed the presence of all three benign lineages of hematopoietic cells. Occurrence of EMH in uterine leiomyoma and intravascular thrombi is very rare. It may be related to systemic hematopoietic stimulation due to severe chronic anemia and local presence of hematopoietic growth factors and/or cytokines.

## 1. Introduction

Extramedullary hematopoiesis (EMH) refers to hematopoiesis outside the bone marrow. It is usually seen in association with hematological diseases. Some common ones include thalassemia, hereditary spherocytosis, sickle cell anemia, congenital dyserythroblastic anemia, and immune thrombocytopenic purpura [1, 2]. Liver, spleen, and lymph nodes are frequently involved [1]. Although uncommon, involvement of other organs or sites, as well as association with nonhematopoietic neoplasms, has also been reported [1, 2]. Tumors reported to be associated with EMH include hemangioma, cerebellar hemangioblastoma, hepatoblastomas, pilomatricoma, hepatic angiosarcoma, endometrial carcinoma, meningioma, hepatic adenoma, spindle cell lipoma, liposarcoma, myofibroblastic tumor, and renal tumors [2]. EMH in uterine leiomyoma and thrombi is very rare. Here we report a case of EMH that is simultaneously present in the stroma of uterine leiomyoma and in the intravascular thrombi within the uterine leiomyoma.

## 2. Case Presentation

A 29-year-old nulliparous female came to the emergency department with heavy vaginal bleeding, hematocrit of 22% (normal range 33–45%), RBC 2.78 × 10^6^/mm^3^ (normal range 3.8–5.2 × 10^6^/mm^3^), WBC 8.5 × 10^3^/mm^3^ (normal range 4–11 × 10^3^/mm^3^), hemoglobin 6.8 g/dL (normal range 11.3–15.2 g/dL), MCV 80 fL (normal range 80–96 fL), MCH 24 pg (normal range 27–33 pg), MCHC 31 g/dL (normal range 32–36 g/dL), RDW 21% (normal range 11–16%), and platelets 531 × 10^3^/mm^3^ (normal range 150–400 × 10^3^/mm^3^). She had a documented history of uterine leiomyoma and menorrhagia for one year. A transvaginal ultrasound confirmed a 10.4 × 9.7 × 9.5 cm mass consistent with leiomyoma occupying fundus and body of the uterus. The patient received multiple blood transfusions due to severe anemia and was treated conservatively with oral contraceptive pills (ethinyl estradiol-norgestimate) due to her desire for fertility. However, she continued heavy vaginal bleeding and remained transfusion dependent. After two months, she developed bilateral upper extremity deep vein thrombosis and multiple superficial vein thromboses. An exploratory laparotomy with uterine myomectomy was performed. Of note, she did not have a history of smoking. She did not have a family history of coagulation disease. The initial coagulation testing revealed normal PT and INR levels and a low PTT (16 seconds). No further coagulation tests for thrombophilia were performed.

**Figure 1:**
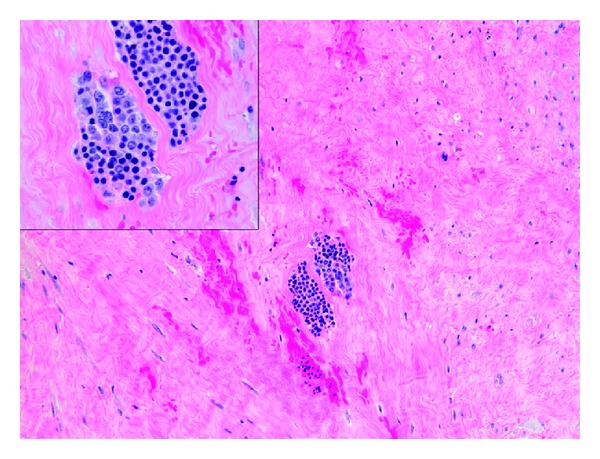
EMH present within smooth muscle of leiomyoma, hematoxylin-eosin stain, magnification 100x (insert 400x).

**Figure 2:**
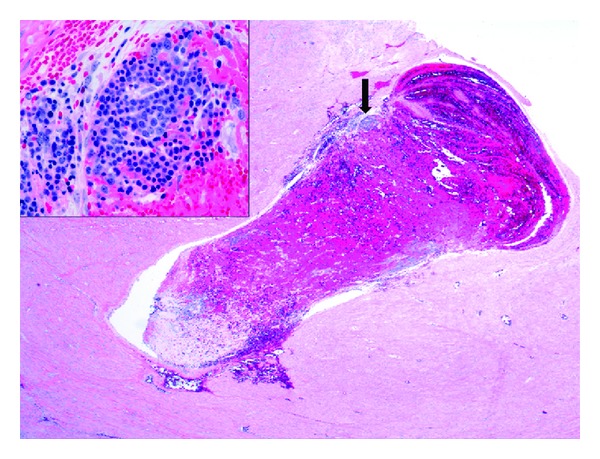
EMH within uterine thrombus, hematoxylin-eosin stain, magnification 100x (insert 400x).

**Figure 3:**
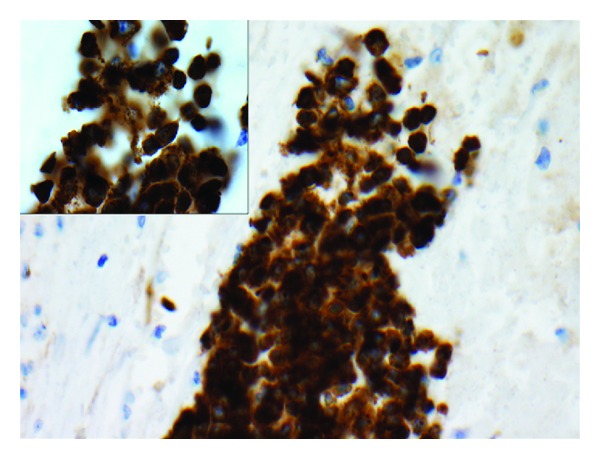
Immunohistochemical stain CD71 shows erythroid precursors, magnification 400x (insert 1000x).

**Figure 4:**
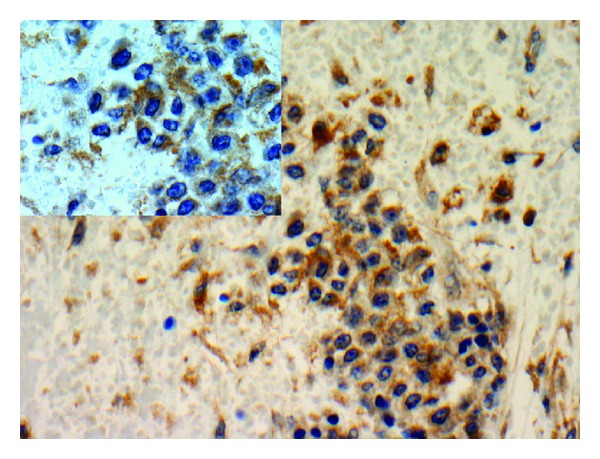
Immunohistochemical stain CD33 shows positive large mononuclear myeloid precursor cells, magnification 400x (insert 1000x).

**Figure 5:**
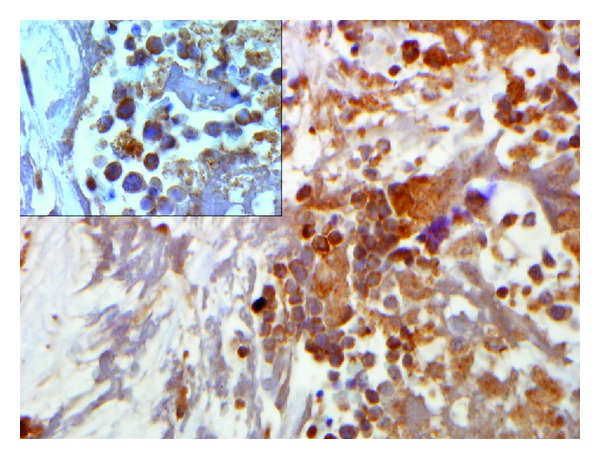
Immunohistochemical stain myeloperoxidase (MPO) shows positive myeloid cells, magnification 400x (insert 1000x).

Gross examination of the specimen revealed a single nodular mass measuring 10.0 × 9.5 × 7.5 cm with a white-tan swirling cut surface. Two hemorrhagic areas were identified measuring 1.0 cm and 1.2 cm in maximum diameter, respectively. Microscopic examination revealed benign smooth muscle tumor consistent with leiomyoma with small cellular aggregates which were further identified as benign hematopoietic precursor cells. The areas consistent with EMH were present within the smooth muscle of leiomyoma (Figure 1). In addition, there were numerous intravascular thrombi, some of which contained hematopoietic precursor cells (Figure 2). EMH foci were not identified within normal myometrium. No necrosis was identified. Immunohistochemical stains were performed to further differentiate the hematopoietic precursor cells. CD43 (L6B, predilute; VT Ventana) and CD71 (10F11, 1 : 160; Leica) demonstrated diffuse positivity consistent with a predominant population of erythroid precursors (Figure 3). Focally positive CD33 (P105441, 1 : 400; Leica), CD34 (QBEnd10, predilute; Dako), CD45 (ZBli(+) PD7/26, predilute; Dako), and myeloperoxidase (predilute; VT Ventana) confirmed the presence of myeloid precursors (Figures 4 and 5). Positive staining for CD61 (202 [ASR], 1 : 100; Leica) demonstrated rare megakaryocytes. Thus, all three lineages of EMH were confirmed.

## 3. Discussion

Extramedullary hematopoiesis in uterine leiomyoma associated with intravascular thrombi has not been previously reported. Leiomyomata are very common benign smooth muscle tumors clinically apparent in about 12–25% of reproductive age women and noted in about 80% of surgically resected uteri [3–5]. However, to our knowledge, only one case of EMH in a degenerating uterine leiomyoma has been reported to date [6]. It was a 66-year-old woman who had a hysterectomy that showed a leiomyoma with degenerative changes and extensive EMH. No evidence of any hematological or systemic disease was found in this patient. Two cases of EMH in thrombi have been reported: a case of a 12-day-old infant with a right middle cranial fossa hematoma and a case of a 78-year-old male with endothelial papillary hyperplasia of the tongue [7, 8].

Normal hematopoiesis depends on a complex interaction of the stem cells with the stroma, cell-cell interactions, and the influence of cytokines and hematopoietic growth factors in the bone marrow microenvironment [2, 9]. It has been proposed that EMH develops when pathophysiologic alterations induce or activate a stem cell niche outside the bone marrow [9].

In our case, multiple factors may play a role. First, the patient had severe chronic anemia due to long-standing heavy vaginal bleeding, which may stimulate hematopoiesis systemically for compensation. Fast turnover in the bone marrow resulted in release of hematopoietic precursors into the blood stream. Second, there may be local stimulation of hematopoietic growth factors and/or cytokines. One possible factor is erythropoietin. Leiomyoma may cause increased erythropoietin production [10]. This has been documented in both uterine and cutaneous leiomyoma [11–13]. In fact, the term “myomatous erythropoiesis syndrome” has been coined for erythropoiesis caused by uterine leiomyoma [14]. Erythropoietin is multifunctional, driving the production, proliferation, and maturation of red blood cell precursors [15]. EMH in a patient with myeloid dysplastic syndrome following administration of erythropoietin has been reported [16]. In addition, factors related to ischemia or tissue injury may also contribute to EMH. We identified numerous thrombi with EMH within the leiomyoma. It is well known that oral contraceptive pills increase the risk of thrombosis [17]. The possible association of oral contraceptive pills and EMH is unknown. The patient developed deep and superficial vein thrombosis after two months of treatment with oral contraceptive pills. The presence of EMH in the thrombi could be explained by the accumulation of circulating hematopoietic precursor cells that were released due to fast bone marrow turnover in the condition of severe chronic anemia. In addition, EMH was previously reported to be present in myocardial infarcts probably related to tissue response to injury [18]. Although EMH has been reported in endometrium, cervix, and uterine isthmus [19–21], our finding of EMH present in both smooth muscle and within the thrombi of uterine leiomyoma is unusual. It may help us to further understand the pathogenesis of EMH. Patient's followup six months after surgery showed normal hematocrit of 37% and resolution of deep and superficial thrombosis of left upper extremity on real-time sonography.
